# Inhibitors of Cyclophilin A: Current and Anticipated Pharmaceutical Agents for Inflammatory Diseases and Cancers

**DOI:** 10.3390/molecules29061235

**Published:** 2024-03-11

**Authors:** Xuemei Zhao, Xin Zhao, Weihua Di, Chang Wang

**Affiliations:** 1 School of Pharmaceutical Sciences, Shandong First Medical University & Shandong Academy of Medical Sciences, Ji’nan 250000, China; zhaoxin202304@163.com (X.Z.); 18363429604@163.com (W.D.); 2 Medical Science and Technology Innovation Center, Shandong First Medical University & Shandong Academy of Medical Sciences, Ji’nan 250000, China

**Keywords:** cyclophilin A, peptidyl-prolyl isomerase, cyclophilin inhibitor, cyclosporin A, cancer, inflammatory diseases

## Abstract

Cyclophilin A, a widely prevalent cellular protein, exhibits peptidyl-prolyl *cis*-*trans* isomerase activity. This protein is predominantly located in the cytosol; additionally, it can be secreted by the cells in response to inflammatory stimuli. Cyclophilin A has been identified to be a key player in many of the biological events and is therefore involved in several diseases, including vascular and inflammatory diseases, immune disorders, aging, and cancers. It represents an attractive target for therapeutic intervention with small molecule inhibitors such as cyclosporin A. Recently, a number of novel inhibitors of cyclophilin A have emerged. However, it remains elusive whether and how many cyclophilin A inhibitors function in the inflammatory diseases and cancers. In this review, we discuss current available data about cyclophilin A inhibitors, including cyclosporin A and its derivatives, quinoxaline derivatives, and peptide analogues, and outline the most recent advances in clinical trials of these agents. Inhibitors of cyclophilin A are poised to enhance our comprehension of the molecular mechanisms that underpin inflammatory diseases and cancers associated with cyclophilin A. This advancement will aid in the development of innovative pharmaceutical treatments in the future.

## 1. Introduction

**Figure 1:**
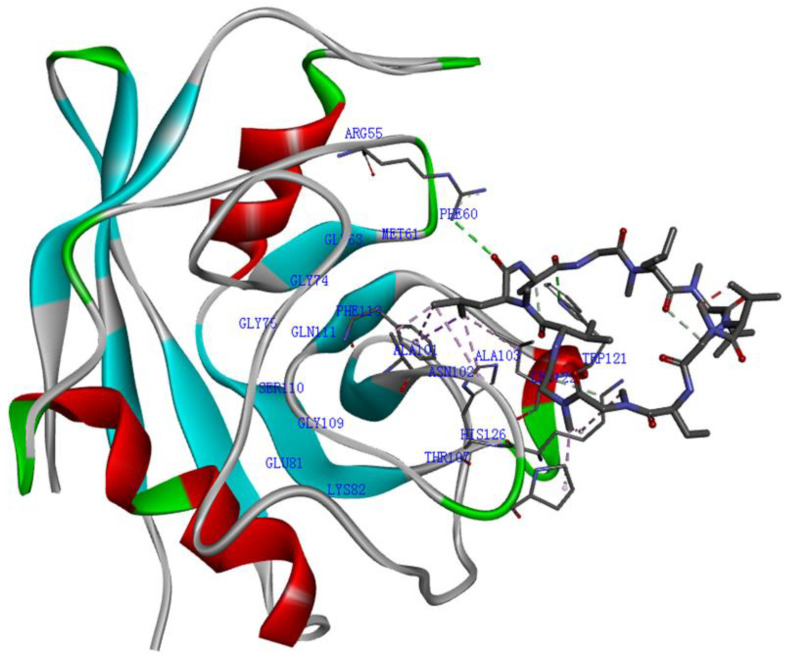
The cyclophilin A–CsA complex. Cyclophilin A binding site (using PDB structure 1CWA). β-sheets in light blue, α-helices in red in cyclophilin A structure. CsA is indicated in black and gray. The 19 residues of cyclophilin A bind in CsA are dark blue.

Cyclophilins are a family of ubiquitously distributed cellular proteins, consisting of at least 16 subtypes in human genome, such as cyclophilin A, cyclophilin B, cyclophilin D, and cyclophilin J [1,2,3,4]. Cyclophilins possess peptidyl-prolyl isomerase (PPIase) activity, which they exhibit by facilitating the *cis*-*trans* isomerization of peptide bonds that precede proline residues, potentially via an electrostatic handle mechanism [3,5,6,7,8]. Among them, cyclophilin A, the prototype of cyclophilins, was first identified and is well characterized [9,10,11]. Cyclophilin A comprises eight antiparallel β-sheets and a pair of α-helices; the active sites of the PPIase in cyclophilin A include Arg55, Phe60, Met61, Gln63, Gly74, Gly75, Glu81, Lys82, Ala101, Asn102, Ala103, Thr107, Gly109, Ser110, Gln111, Phe113, Trp121, Leu122, and His126 (Figure 1) [7,12,13,14,15]. These binding sites can be occupied by cyclosporin A (CsA), which is a potent cyclophilin A inhibitor and one of the powerful immunosuppressive drugs, and is the most extensively studied and strongest binding ligand of cyclophilin A [7,10,12,13,15]. Most of these structure features are evolutionarily conserved in the homologues of cyclophilins [7].

Cyclophilin A is identified as a critical component in various biological processes and associated diseases, such as protein folding/trafficking, immune responses, cell signaling, vascular disease pathogenesis, viral infections, rheumatoid arthritis, atherosclerosis, and cancer development [9,16,17,18,19,20,21,22,23,24,25,26,27,28,29,30,31,32,33,34,35,36,37,38]. Although the underlying mechanisms remain to be further investigated, they are possibly multifaceted and indeed distinct regarding intracellular and extracellular cyclophilin A proteins. Regarding intracellular cyclophilin A, it predominantly acts through the PPIase-dependent and/or -independent protein–protein interaction [18,26,39,40]. Cyclosporine A (CsA), known for its potent inhibition of cyclophilin A and significant immunosuppressive properties, is well characterized, it binds to cyclophilin A, inhibiting its PPIase activity; subsequently, the cyclophilin A–CsA complex interacts with and inhibits calcineurin, it acts as a calcium/calmodulin-activated serine/threonine-specific protein phosphatase, which obstructs the translocation of nuclear factor of activated T-cells (NF-AT) from the cytosol to the nucleus, ultimately inhibiting T-cell activation [41,42,43]. Most recently, a novel intracellular target of cyclophilin A, the inosine-5′-monophosphate dehydrogenase 2, has been identified, which can bind with the complex of cyclophilin A–sanglifehrin A (SFA, an inhibitor of cyclophilin A, described below) to modulate cell proliferation and eventually inhibit T-cell activation induced by SFA [44]. In addition, a plenty of evidence indicates that intracellular cyclophilin A plays a crucial role in the lifecycle of various viruses, including HIV-1, influenza, hepatitis B and C, vesicular stomatitis virus, vaccinia virus, SARS-CoV, nidovirus, feline and porcine CoV, and rotavirus (RV), via its binding with capsid or nonstructure viral proteins [28,29,36,45,46,47,48,49,50]. Furthermore, cyclophilin A is notably overexpressed in numerous human cancers and cancer-related cell lines [24,25,31,39,51,52,53,54,55,56,57,58,59]. Although its biological roles in tumor cells remain elusive, cyclophilin A may enhance cell survival under stressful conditions, such as those associated with the proliferation of signaling proteins, antiapoptotic proteins, transcription factors, or cell migration regulatory proteins, including CYCS (cytochrome c, somatic) [60], ITK (interleukin-2 inducible T-cell kinase) [61], ASK1 (apoptosis signaling-regulating kinase 1) [40], and CRKII (CT-10 (a kind of avian virus) regulator of kinase) [39]. For instance, cyclophilin A can bind adaptor protein CRKII to sterically restrict the accessibility of CRKII Tyr221 to its kinase ABL1 (Abelson murine leukemia viral oncogene homolog 1) or EGFR (epidermal growth factor receptor), which thereby, inhibits CRKII phosphorylation and keeps it at the active form and enhances CRKII-mediated signaling to promote tumor cell migration [39]. Altogether, these findings indicate that intracellular cyclophilin A functions at multiple levels, including its critical roles in immune response, viral infection, and tumorigenesis.

In addition to its predominant cytosol localization, cyclophilin A can be secreted by cells in response to inflammatory stimuli like hypoxia, infection, and oxidative stress [21,32,62,63,64,65]. A body of evidence by in vitro studies and in vivo genetically modified mouse models demonstrates that extracellular cyclophilin A is involved in inflammatory diseases such as viral infection [30,45,66,67,68,69], periodontitis [70], and atherosclerosis [69] by means of promoting cell chemotaxis and cell migration (especially leukocyte chemotaxis), and eventually enhancing inflammation [32]. The function of extracellular cyclophilin A is mainly mediated by the “extracellular matrix metalloprotease inducer” (EMMPIN, also called CD147) [71]; this protein is a broadly expressed plasma membrane protein found in various cells, including hematopoietic, epithelial, and endothelial cells [72]. The cyclophilin A–CD147 complex initiates a signaling cascade, stimulating cell proliferation and chemotaxis through activation of MAPK pathways, including ERK1/2 and p38 MAPK [51,59,73]. For example, extracellular cyclophilin A was reported to be essential for vascular remodeling, as demonstrated by CyPA^−/−^ mouse model [64,74,75], and mounting evidence has highlighted its potential effect in atherosclerosis, which is a complicated, progressive inflammatory disease [30]. Altogether, these studies suggest that cyclophilin A drives cellular functions not only via its chaperone and PPIase activity but also through cyclophilin A-directed signal transduction in inflammatory diseases and human cancers.

Considering that cyclophilin A’s important roles in inflammatory diseases and cancers, cyclophilin A-based targeting will be beneficial to conquer these disorders. As such, cyclophilin A has attracted considerable attention because of its potential use as a therapeutic target based on its PPIase activity. Cyclosporin A (CsA), the first potent cyclophilin A inhibitor, over three decades ago, marked a new epoch in organ transplantation [76,77]. Although CsA is an old medicine in the treatment of diseases, research on CsA has never stopped. These studies involve the role of CsA in cancer therapeutics [55], the combination use of CsA in reducing resistance to chemotherapeutic drugs [78] and toxic side effects [79] of antitumor drugs, the synthesis and discovery of CsA derivatives and structural analogues, the development of new functions of CsA [80,81], and identification of new prognostic biomarkers [82], which have always been a hot research topic. Since then, numerous cyclophilin A inhibitors have emerged and been characterized. In the present review, we discuss available data about cyclophilin A inhibitors, including cyclic peptides, peptide analogues, and other small molecule compounds, and outline the most recent advances in clinical trials involving these agents. The exploration and refinement of cyclophilin A inhibitors are expected to deepen current understanding of the molecular mechanisms of these diseases and aid in the development of new pharmaceutical treatments soon.

## 2. Cyclic Peptides as Cyclophilin A Inhibitors

### 2.1. Cyclosporin A (CsA)

CsA (Compound **1**, C1) (Table 1), an acyclic undecapeptide, was first discovered by Jean-Francois Borel in 1976 [83], and approved as a potent immunosuppressant drug by U.S. Food and Drug Administration (FDA) in 1983. CsA inhibits the PPIase activity associated with the broad family of cyclophilins. Acting as an inhibitor of cyclophilin A, CsA suppresses the activity of calcineurin, a calcium/calmodulin-activated serine/threonine protein phosphatase, which abolishes dephosphorylation-dependent nuclear translocation of transcription factor NF-AT, and ultimately suppresses T-cell activation [84,85]. Therefore, CsA exhibits profound inhibitory effects on immunity, and has been extensively used for immune suppression in allogeneic transplantation of bone marrow/hematopoietic stem cells and solid organs for prevention and treatment of graft-versus-host diseases, and it also plays a role in various inflammatory conditions, such as rheumatoid arthritis and psoriasis [86]. Also, based on the high level of cyclophilin A in human malignancies, CsA has been used to target human cancers, either alone or in combination with other agents [87,88]. However, there are two possible issues for CsA. For one, the clinical use of CsA has shown that it may be not a perfect drug because of its poor aqueous solubility and serious side effects, including hepatotoxicity, nephrotoxicity, hyperkalemia, hyperuricemia, renal dysfunction, leukopenia, lymphoma, or skin cancer [89,90,91,92]. At this point, more efforts are needed to reduce the toxicity and the off-target effects of CsA in clinic [93]. For another, since CsA has also been used in inflammatory diseases, one future direction is to find nonimmunosuppressive CsA derivatives or novel agents to inhibit the role of cyclophilin A in inflammation but not affect host immunity during viral infection control or cancer treatment.

Other research shows that CsA decreases SARSCoV-2 replication in vitro and decreases mortality rates of coronavirus disease 2019 (COVID-19) patients. The research found that the nucleocapsid protein significantly depends on cyclophilin A, and identified the docking sites of nucleocapsid with cyclophilin A [94]. Laurie et al. demonstrated the nucleocapsid as a potential indirect therapeutic target of CsA, which may impede coronavirus replication by obstructing nucleocapsid folding [95].

In the latest study, researchers compared the effects of CsA and direct-acting antivirals (DAAs), which consist of inhibitors of nonstructural proteins 5A (NS5A), NS3/4A, and NS5B in Huh7.5.1 cells. The results showed that CsA inhibits HCV infection at the same speed as the NS5A and NS3/4A inhibitors of DAAs. It has been reported that DAAs are the fastest antiviral drugs in inhibiting HCV infection. This study further elucidates that CsA can rapidly inhibit the level of infectious extracellular viruses, but it has no significant effect on intracellular infectious viruses [96].

### 2.2. Cyclolinopeptides and the Analogues

**Figure 2:**
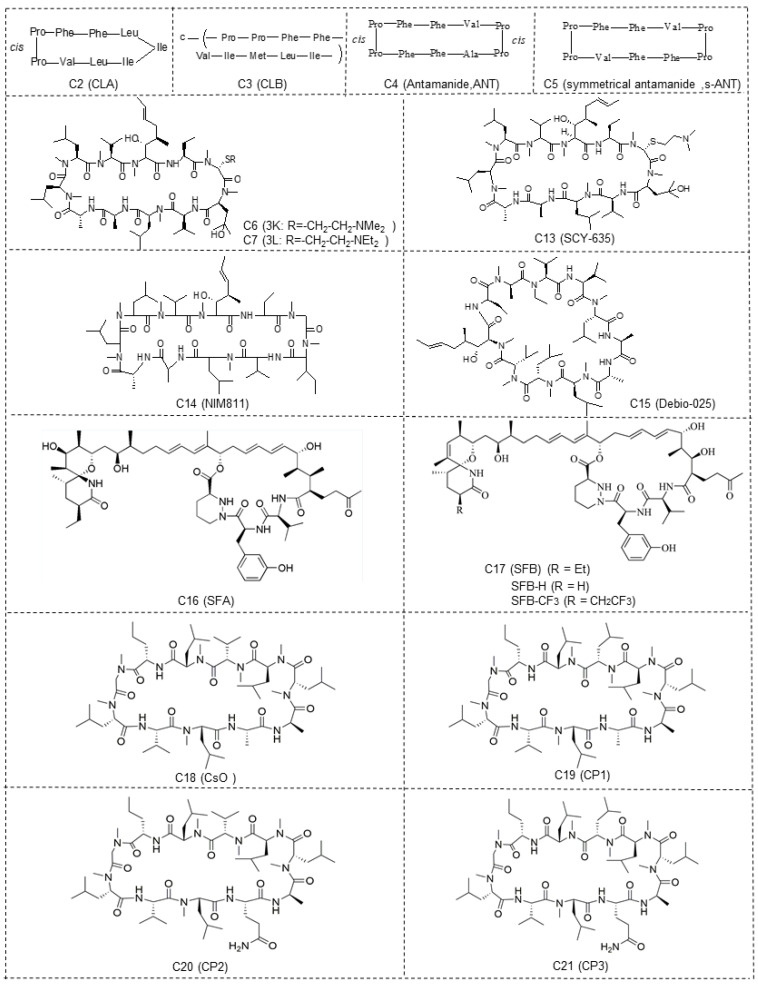

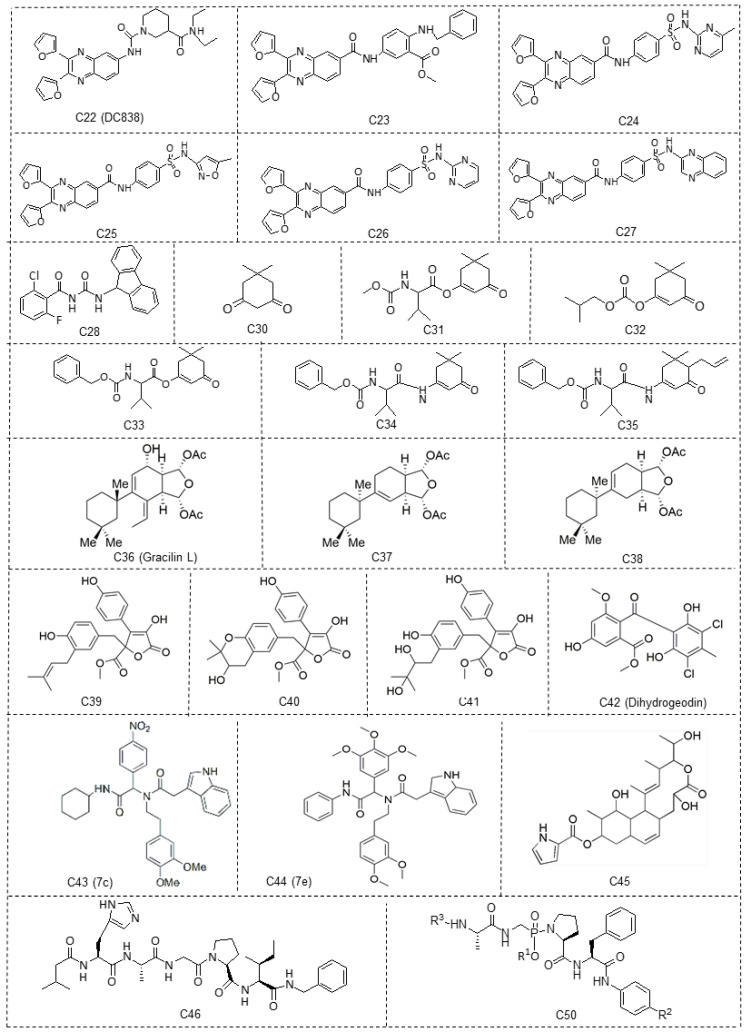
Chemical structure of the major compounds as inhibitors of cyclophilin A.

Cyclolinopeptide A (CLA, C2) (Figure 2) is a homodetic cyclic nonapeptide (an analogue of antamanide), which was isolated from linseeds [97]. Its bioactive conformation was presumably attributed by the sequence Pro-Pro-Phe-Phe. The initial recognized biological activity of CLA was its capacity to inhibit the hepatocyte transport system used for bile salts, ethanol, and cysteamine, as well as dimethylsulfoxide [98]. Importantly, CLA was reported to bind to cyclophilin A and other cyclophilins, and the cyclophilin A–CLA complex inhibited calcineurin-dependent T-cell activation, showing the similar mechanism of immunosuppressive effect to CsA [99]. However, CLA has a tenfold lower affinity for calcineurin compared to CsA and is considered nontoxic [100,101,102,103]. In addition to CLA, another cyclic nonapeptide found in linseeds is cyclolinopeptide B (CLB, C3) (Figure 2), which differs from CLA in its amino acid composition and sequence. CLB is characterized as a more potent suppressor of the effector phase of delayed-type hypersensitivity reactions than CLA [104]. The other natural cyclic peptides (C4 and C5) (Figure 2) show structural similarities with CLA, both of which bind with bovine cyclophilin A and exhibit lower immunosuppressive activity than CLA [102]. All cyclolinopeptides and their analogues form complexes with cyclophilins and can, in this state, inhibit the phosphatase activity of calcineurin [101,102,105], indicating that cyclolinopeptides might be promising inhibitors of cyclophilin A.

### 2.3. Cyclosporin A Derivatives

Recently, Evers and colleagues reported a sort of novel CsA derivatives, [2-(dimethyl or diethylamino)-ethylthio-Sar]^3^-[(4′-OH)MeLeu]^4^-CsA 3K (C6) and 3L (C7) (Figure 2) [106]. These CsA derivatives display potent anti-HIV-1 activity (IC_50_: ~46 nM) in vitro, while exhibiting low immunosuppressive capacity (IC_50_: ≥1500 nM) [106]. Thus, these derivatives could serve as novel and promising candidates for treating HIV-1 infection and may be effectively combined with other anti-HIV-1 drugs.

### 2.4. Cyclosporin A Analogues

Wei and colleagues reported a line of CsA analogues, which were modified by a solution-phase fragment coupling strategy [107]. The analogues modified at position 4 are the inhibitors for cyclophilin rotamase as potent as CsA, but lose immunosuppressive activity; the analogues modified at position 8 also exhibit threefold lower inhibitory rotamase activity of cyclophilins than CsA. Meanwhile, the saturated dihydro-CsA, altered in its binding domain, exhibits only one-fifth the potency of CsA in inhibiting cyclophilin rotamase activity. Additionally, the dehydro Ala8-CsA analogue does not inhibit T-cell proliferation at concentrations up to 10 µM, indicating that maintaining a *d*-configuration at this position is crucial for calcineurin phosphatase inhibition. Moreover, there are other analogues of CsA (C8–C12), which are illustrated in Table 1. These analogues’ ED_50_ are around 100 nM, slightly higher than that of CsA (5 nM) [107]. Collectively, these findings suggest that the mentioned CsA analogues are more likely to inhibit cyclophilin rotamase without exerting immunosuppressive effects.

CsA has a immunosuppressive effect through binding to calcineurin, especially by the position 4 (P4), P5, and P6 side chains of CsA. In order to improve the inhibitory effect of CsA derivatives on cyclophilin A and reduce its immunosuppressive effect, CsA derivatives have been modified by changing the P3 side chain and substituting P4, P5, and P6 side chains; these modifications can increase CsA derivatives binding to cyclophilin A [49].

4MCsA, an albumin-bound CsA analogue, presents prospective inhibitory effects on chemotactic activity and inflammation by targeting extracellular cyclophilin A. The binding affinity of 4MCsA to cyclophilin A is similar to that of CsA, but lacks immunosuppressive ability and cytotoxicity [108].

#### 2.4.1. SCY-635

SCY-635 (C13) (Figure 2) is a novel CsA-based analogue that does not cause immunosuppression and effectively suppresses HCV replication in vitro [109]. It inhibits cyclophilin A’s PPIase activity at nanomolar levels, but shows no perceptible inhibitory effect on the phosphatase activity of calcineurin under the concentrations up to 2 µM. Additionally, SCY-635 does not induce major cytochrome P450 enzymes 1A2, 2B6, and 3A4, and is a weak inhibitor and a poor substrate for P-glycoprotein, suggesting that it may have less potential drug–drug reaction. SCY-635 also shows synergistic antiviral activity with interferon-alpha 2b (IFNα-2b) and additive antiviral activity with ribavirin [110,111]. Therefore, SCY-635 might be a promising novel antiviral agent with an acceptable safety profile during treatment.

#### 2.4.2. [Me-Ile-4]cyclosporine (NIM811)

[Me-Ile-4]cyclosporin (NIM811), i.e., *N*-methyl-L-isoleucine-cyclosporin (C14) (Figure 2), is an analogue of cyclosporine substituted at position 4 with *N*-methyl-L-isoleucine, which is isolated from the fungus *Tolypocladiumniveurn* [112]. NIM811 is a first cyclosporin-based nonimmunosuppressive inhibitor of cyclophilins. In contrast to CsA, NIM811 lacks immunosuppressive effectiveness while fully retaining its binding capacity to cyclophilins [112]. NIM811 exhibits a higher affinity for all cyclophilins compared to CsA [113], which makes it a powerful suppressor for viral replication since cyclophilins, including cyclophilin A, are involved in the formation of viral particles by interacting with capsid proteins of a series of viruses such as HIV-1 and HCV [112,113,114,115,116,117,118,119]. In addition to binding to cyclophilins, NIM811 can interact with the components of protein/lipid trafficking and spliceosome pathway, which in turn contributes to the inhibition of viral replication and particle formation [113]. Importantly, it causes a smaller degree of nephrotoxicity than CsA, together indicating that it is possibly a better CsA analogue for virus treatment [112].

Regarding its potential role in inflammation, e.g., in coxsackievirus B3-induced myocarditis, NIM811 can result in low level of metalloproteinase-9 and a reduction in inflammatory lesions, represented by the extent of lesion area is significantly decreased at 28 days post-infection compared to that at 8 days post-infection when treated with NIM811 [120]. Therefore, NIM811 represents a novel promising inhibitor of cyclophilin A for inhibiting viral infection and inflammation, but not acting as a potent immunosuppressant agent.

In addition, NIM811 can induce apoptotic cell death of human and murine melanoma cells. It may trigger apoptosis through transient mitochondrial depolarization, which leads to the efflux of proteins from the intermediate space, including cytochrome c, procaspase 9, apoptosis-inducing factor, and endonuclease G, sufficient to trigger “apoptosome” formation and initiate the execution phase of apoptosis [121]. However, NIM811 also has a cytoprotective effect by inhibiting mitochondrial permeabilization transition pore (mPTP) opening to prevent in situ mitochondrial inner membrane permeabilization and depolarization [122,123,124,125,126,127], although this cytoprotective effect depends on its binding to cyclophilin D, but not cyclophilin A [125,128]. As such, NIM811 demonstrates a complicated role in regulation of cancer cell growth. 

In addition, NIM811 can effectively inhibit the replication of HCoV-229E. CsA and NIM811 derivatives block the interaction between cyclophilin A and nucleocapsid proteins, indicating the mechanism by which cyclophilin A inhibitors inhibit virus replication [129].

#### 2.4.3. Alisporivir (Debio-025) 

Debio-025 (C15) (Figure 2) is a CsA analogue that significantly disrupts the lifecycle of the hepatitis C virus [130,131] and the replication of HIV-1 [132]. Debio-025 differs from CsA by having an additional methyl group at position 3 of the cyclic undecapeptide and an *N*-ethylvaline instead of an *N*-methylleucine at position 4. Unlike CsA, Debio-025 does not exhibit immunosuppressive activity in vitro and in vivo. The structure of the cyclophilin A-Debio-025 complex, hindered by steric interference with calcineurin’s Val4 residue, contrasts with the cyclophilin A–CsA–calcineurin ternary complex, where the Leu4 side chain fits into a hydrophobic cavity at the calcineurin interface. This provides a rational basis for Debio-025’s nonimmunosuppressive properties [133].

Furthermore, Debio-025 inhibits cell migration. When administered in vivo in a triple-negative breast cancer in situ model, Debio-025 alone or in combination with anti-PD-1 mAb shows antitumor efficacy, reducing tumor volume and metastatic lung dispersion. In addition, when analyzed by NanoString immunoassay, treating Debio-025 with anti-PD-1 mAb increased T-cell signaling and innate immune signaling in the tumor microenvironment [134].

### 2.5. Sanglifehrin A (SFA)—A Natural Product

A new class of compounds named sanglifehrins has been identified through screening of the cyclophilin-binding substances from microbial broth extracts of *Streptomyces* sp. A92-308110 [135,136]. Among 20 different sanglifehrins isolated, sanglifehrin A (SFA, C16) (Figure 2) is the most abundant. Its affinity for cyclophilins is about 60 times higher than CsA in a cell-free competitive binding assay (IC_50_ = 6.9 ± 0.9 nM for SFA vs. IC_50_= 420 ± 56 nM for CsA) [137]. The chemical and three-dimensional structure of SFA greatly differs from CsA, suggesting distinct mechanisms in its immunosuppressive capacity [138]. More surprisingly, SFA’s complex macrocyclic structure, featuring a unique tripeptide, an (*E,E*)-diene unit, and a polypropionate section, results in an exceptionally strong affinity for cyclophilins [139]. Furthermore, SFA also shows significant immunosuppressive activity in the murine mixed lymphocyte reaction (IC_50_ = 170 nM) [137], and has an inhibitory effect on T-cells [140,141,142] and dendritic cells [143,144]. For instance, SFA remarkably abrogates production of bioactive IL-12p70, the major producer of IL-12 secreted by dendritic cells, to inhibit the activity of dendritic cells [143]. These studies indicate that SFA is a novel and potent immunosuppressant agent. 

Based on the SFA structure as the lead structure, the macrocycle was simplified and some cyclophilic protein inhibitors were synthesized. Schiene-Fischer et al. and Han et al. have summarized this [38,49]. Now, the total synthesis of sanglifehrin A and sanglifehrin B (SFB, C17) (Figure 2) and preparation of additional analogs have been achieved. Their biological activity has been evaluated in Jurkat cells and they can also stabilize protein–protein interactions [145].

### 2.6. Cyclosporin O (CsO)—A Natural Macrocycle

Cyclosporin O (CsO, C18) (Figure 2) and its derivatives (CP1-3, C19–C21) (Figure 2) are macrocyclic peptides with structural diversity and more rational design. In nonpolar media, CsO exhibits a conformation similar to CsA. CsO exhibits its own characteristics; for example, it has a higher plasma concentration than CsA, due to its minimal binding to cyclophilin A, lower accumulation in red blood cells, and moderate oral bioavailability (F = 12%) [146].

## 3. Small Molecular Inhibitors of Cyclophilin A

### 3.1. Quinoxaline Derivatives

Li and colleagues identified a novel quinoxaline derivative, 2,3-di(furan-2-yl)-6-(3-*N,N*-diethyl-carbamoyl-piperidino) carbonylamino quinoxaline (DC838, C22) (Figure 2) as a potent inhibitor against human cyclophilin A [147]. Its IC_50_ for cyclophilin A is 0.41 µM, as determined by PPIase activity assay. The *K*_D_ value of the cyclophilin A-DC838 complex is 7.09 µM, and the *K*’_D_ value 3.78 µM, as analyzed by surface plasmon resonance and fluorescence titration techniques. In vivo studies also revealed that DC838 inhibits mouse spleen cell proliferation induced by concanavalin A. In addition, the specific binding site of DC838 to cyclophilin A has been elucidated by using molecular docking simulation at the atomic level, providing useful information in discovering the novel immunosuppressors based on quinoxaline derivative [147].

Meanwhile, another report identified sixteen novel small molecule inhibitors of cyclophilin A, also belonging to quinoxaline derivatives. This discovery was made using a strategy that integrates focused combinatorial library design, virtual screening, and chemical synthesis [148]. These molecules bind to cyclophilin A with binding affinities (*K*_D_) ranging from 0.076 to 41.0 µM, and five of them (C23–C27) (Figure 2) are the potent cyclophilin A PPIase inhibitors with IC_50_ values of 0.25–6.43 µM. Therefore, these novel chemical entities could serve as leads for developing new therapies targeting the cyclophilin A pathway in immune or cancer cells.

### 3.2. Cyclophilin A Inhibitor 239836

Compound **239836** (C28) (Figure 2) acts as an inhibitor of cyclophilin A (IC_50_ = 1.5 nM), which is approximately 27-fold more potent than CsA, as determined by in vitro assays [149]. The chemical formula of this compound is C_21_H_14_ClFN_2_O_2_. Moreover, C28-treated non-small-cell lung cancer cell line 95C showed that metalloproteinase-9 activity is significantly decreased in a dose-dependent manner, which is a result of suppression of cyclophilin A induced by C28 [149]. This compound is still under development.

### 3.3. Aryl 1-Indanylketones

**Figure 3:**
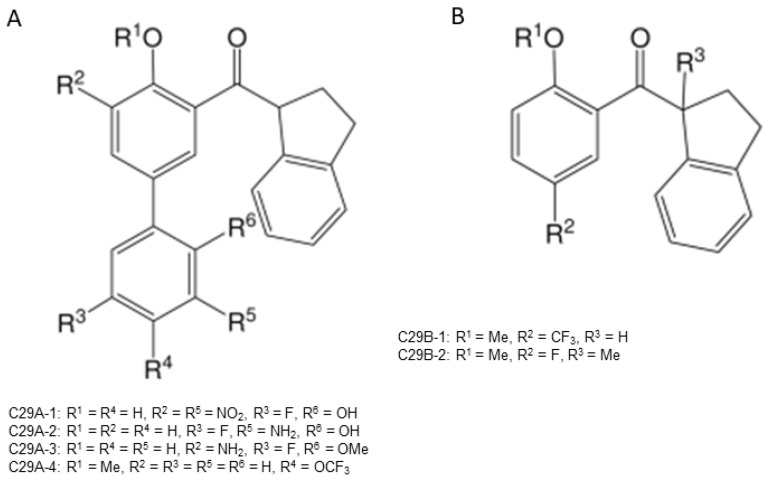
Chemical structure of aryl 1-indanylketones (C29) and their derivatives. (**A**): The nucleus of biaryl indenyl methanone, where R^1^-R^6^ can be substituted by different groups to generate C29A1-4. (**B**): The nucleus of aryl indenyl methanone, where R^1^-R^3^ can be substituted by different groups to generate C29B1-2.

A novel pair of small molecule inhibitors of cyclophilins, i.e., C29A and C29B, has been identified on the basis of aryl 1-indanylketones, which is capable of discriminating between cyclophilin A and cyclophilin B in vitro (Figure 3). The binding of cyclophilin A to the inhibitor C29A has been characterized through fluorescence-anisotropy-based and isothermal titration calorimetry-based cyclosporin competition assays. These inhibitors specifically impair cyclophilin A- but not cyclophilin B-mediated chemotaxis of mouse CD4^+^ T-cells, providing in vivo biological proof of selectivity [150]. The derivative of this inhibitor, C29A-1, enhances selectivity for cyclophilin A over other cyclophilins, especially in the case of cyclophilin B; this inhibitor maintains the highest discriminatory ability between cyclophilin A and B, exceeding a factor of 200. However, among the aryl 1-indanylketone series, the most active inhibitor of cyclophilin A is C29A-2 (*K*_I_ = 0.3 ± 0.1 μM), which, meanwhile, inhibits cyclophilin B with a *K*_I_ of 12 ± 5 μM, thereby discriminating between cyclophilin A and cyclophilin B by a factor of 40. In addition, the two enantiomers of C29B-2 were also analyzed. The inhibitory (*R*)-enantiomer demonstrates a 40-fold selectivity for cyclophilin A, whereas the (*S*)-configuration at the 1-methyl position completely negates inhibition of both cyclophilin A and B [150,151]. 

### 3.4. Dimedone Analogues

The dimedone family of cyclophilin inhibitors, including C30–C35 (Figure 2), has been found, which was achieved using the database-mining program LIDAEUS and in-silico screening techniques. These dimedone analogues display a consistent “ball and socket” binding mode, with a dimethyl group occupying the hydrophobic binding pocket of human cyclophilin A, akin to the interaction of the natural inhibitor CsA [152]. The most potent derivative, C35, binds to cyclophilin A with a *K*_d_ of 11.2 ± 9.2 µM. Its IC_50_ for inhibiting cyclophilins in *C. elegans* is 190 µM, significantly higher than CsA’s 28 µM. These dimedone analogues offer a novel framework for synthesizing peptidomimetic molecules with potential efficacy against cyclophilins and related inflammatory diseases [152,153].

### 3.5. Gracilins—Natural Diterpenes Derivative

Gracilins is a diterpenoid compound isolated from the marine sponge *Spongionella gracilis*. Natural gracilins and synthetic derivatives have shown affinity with cyclophilic proteins. Gracilin L C36 (Figure 2) and two synthetic analogues, compounds **1** and **2** (C37–C38) (Figure 2), have shown anti-inflammatory effects in a cellular model of inflammation. CsA is used as a control, and these compounds can reduce the expression of inflammatory mediators and target proteins, and activate antioxidant mechanisms under inflammatory conditions. Therefore, natural and synthetic gracilins have the potential to be developed into anti-inflammatory drugs [154].

### 3.6. Dichloro-Benzophenone Derivative—Natural Compound

In addition to butyrolactone I (C39), V (C40), and VI (C41) (Figure 2), dichloro-dibenzophenone derivatives, including dihydrogeodin (C42) (Figure 2), were also extracted and isolated from the thermophilic fungus *Aspergillus terreus* TM8. Using 1D, 2D NMR, and ESI HR mass data, as well as X-ray crystallography, researchers reported the structure of dihydrogeodin (C42). The docking and molecular dynamics simulation of dihydrogeodin with isomerase cyclophilic A showed its important prospective activity as an antiviral and immunosuppressive factor [155].

### 3.7. Other Novel Small Molecular Inhibitors of Cyclophilin A

Recently, 12 bisamide compounds were designed and synthesized, and their anti-HCV activity and cytotoxicity were tested. Among them, the bisamide derivative 7c (C43) (Figure 2) is a promising compound with strong anti-HCV activity at subtoxic concentrations. The EC_50_ value of 7c is 4.2 ± 0.1 µM. The CC_50_ value of 7c is greater than 100 µM. The study of molecular docking indicates that 7c is located at the active site of cyclophilin A. In addition, 7c was directly bound to cyclophilin A by surface plasmon resonance (SPR) experiments. All these studies suggest that derivative 7c is a potent cyclophilin A inhibitor [156]. 

In another article, 16 bisamide derivatives were designed and the binding mode for cyclophilin A was switched. Docking research has shown that 7e (C44) (Figure 2) is located in the gatekeeper pocket, with a selectivity index exceeding 18.9. The EC_50_ value of 7e is 5.3 μM, but at 100 μM, it has no cytotoxicity. The SPR results indicate that 7e can bind with cyclophilin A, with a *K*_D_ of 3.66 μM. 7e as a cyclophilin A inhibitor can serve as an alternative anti-HCV drug in future combination therapy [49]. 

There are also many studies on new nonpeptide small molecular cyclophilin inhibitors. They exhibit potent in vitro PPIase inhibitory activity and antiviral activity against hepatitis C virus, human immunodeficiency virus, and coronaviruses [157,158]. 

The latest research has found that 23-demethyl 8,13-deoxygenicin (C45) (Figure 2), a natural inhibitor of cyclophilin A, either as monotherapy or in combination with afatinib, can inhibit the growth of cancer stem cells in non-small-cell lung cancer by disrupting the crosstalk between cyclophilin A/CD147 and EGFR. Its mechanism of action is that C45 can inhibit proliferation and lead to apoptosis of MKN45 gastric cancer stem-like cells by regulating the cyclophilin A/CD147-mediated signaling pathway [159,160].

## 4. Peptide Analogues

### 4.1. Heptapeptides

Based on the X-ray structure of Gag fragments: cyclophilin A complexes, Li [161] generated 52 modified peptides to explore the interaction determinants of the complex and to identify peptidic ligands with higher affinity than the capsid domain of the Gag protein. Among these peptides, the presence of an *N*-terminal valine or substitution of the C-terminal alanine amide with a benzylamide group (-NHBn) enhances high-affinity binding. The combination of both modifications results in a highly potent competitor, Dav-His-Ala-Gly-Pro-Ile-NHBn (Dav, deaminovaline; NHBn, benzylamine) (C46) (Figure 2). This competitor exhibits a stronger affinity for cyclophilin A (*K*_d_ = 3 ± 0.5 μM) than the entire capsid protein (*K*_d_ = 16 ± 4 μM), and has a very low affinity for FKBP12, another important PPIase in the immunophilin family. These studies suggest that the title compound is not a substrate of cyclophilin A, but interacts preferentially in the *trans* conformation for immune suppression.

### 4.2. N- or C-Terminal Modification of Gag Peptide

A study employing molecular docking and 3D-QSAR approaches investigated 22 Gag peptide analogues interacting with human cyclophilin A [162]. The Lamarckian Genetic Algorithm (LGA) and divide-and-conquer methods were applied to determine the binding orientations and conformations of these peptide analogues with cyclophilin A. Among these analogues, the peptides C47 (Dav-His-Ala-Gly-Pro-Ile-Ala-NH_2_), C48 (Dav-His-Ala-Gly-Pro-Ile-NH-CH_2_-Ph), and C49 (Dav-His-Ala-Gly-Pro-Acp-NH-CH_2_-Ph) (Dav, deaminovaline; Acp, 2-aminocyclopentanecarboxylate) were identified based on a novel interaction model. The *N*-termini of compounds **C47**, **C48**, and **C49** were modified by the addition of a deaminovaline group. Meanwhile, the C-termini of C48 and C49 were modified by the addition of a benzyl group (-Ph). These new peptide analogue inhibitors exhibit much higher inhibitory activities for cyclophilin A [162].

### 4.3. Trp-Gly-Pro (WGP)

Another study using the Miyazawa–Jernigan matrix and the hidden Markov model identified a peptide, Trp-Gly-Pro (WGP), acting as an inhibitor for cyclophilin A and FKBP12 [163]. This peptide, though smaller in molecular weight than CsA, binds to cyclophilin A with a similar affinity, having a dissociation equilibrium constant *K*_D_ of 3.41 × 10^−6^ M, which is in the same order as CsA (*K*_D_ = 6.42 × 10^−6^ M). Also, WGP inhibits cyclophilin A-mediated PPIase activity with IC_50_ values of 33.11 nM and 10.25 nM, respectively. In addition, this peptide also inhibits HIV-1 infection and exhibits lower toxicity and better oral availability and solubility than CsA, making it a potential CsA replacement in clinical applications [164].

### 4.4. Pseudopeptides

Demange [165] inserted the Gly*ψ* (PO_2_R^1^-N) Pro motif (R = alkyl or H) into Suc-Ala-Ala-Pro-Phe-*p*NA (*p*NA, *p*-nitroaniline), a peptide substrate of cyclophilin A to create a pseudopeptide Suc-Ala-Gly*ψ* (PO_2_Et-N) Pro-Phe-*p*NA (C50) (Figure 2). This pseudopeptide binds to cyclophilin A with a *K*_d_ = 20 ± 5 μM and selectively inhibits the cyclophilin A’s PPIase activity at IC_50_ = 15 ± 1 μM. This pseudopeptide does not inhibit the PPIase activity of FKBP12, making compound **C50** a novel transition-state mimic inhibitor of cyclophilin A. 

### 4.5. “Self-Reproduction of Chirality” Analogues

**Figure 4:**
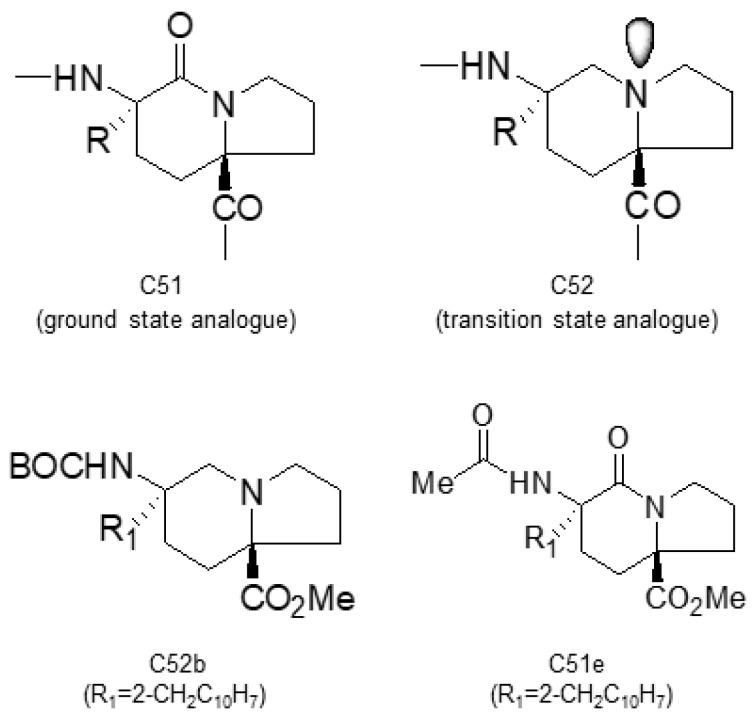
Chemical structure of the compounds **C51** and **C52** based on their ground or transition states.

Based on the structures of proline-containing peptides [166], both ground-state analogues (C51) and transition-state analogues (C52) were prepared. While C52 shows minimal binding to the active site (*K*_d_ = 77 μM for C52b), several ground-state analogues exhibit low micromolar affinity (*K*_d_ = 1.5 μM for C51e) (Figure 4), suggesting their potential as lead compounds for cyclophilin A inhibitors.

## 5. Cyclophilin A Inhibitors in Clinical Trials 

Cyclophilin A is implicated in many human disorders, including inflammatory diseases such as viral infection and atherosclerosis, and cancers [9]. As described above, more and more inhibitors of cyclophilin A have been identified and tested for treating these diseases. Although CsA, the potent inhibitor of cyclophilin A, has been approved as a potent immunosuppressive drug by the U.S. FDA for over three decades, many other inhibitors are not approved yet. It is delighting, however, that a line of cyclophilin A inhibitors have been entered into clinical trials (Table 2). Based on the potent role of cyclophilin A inhibitors in preventing graft-versus-host immunity or rejections, these clinical trials mainly focus on immunosuppression after liver or kidney transplantations or hematological stem cell transplant after bone marrow failure or leukemia/lymphoma. Clinical trials of CsA, both alone and in combination with other agents, are being undertaken to reduce its severe side effects (especially CsA-induced skin cancer), or to investigate the optimal regime in organ transplantation, and to detect the efficacy of antiviral infection. For example, the efficacy and toxicity of CsA and irinotecan hydrochloride in the treatment of advanced colorectal cancer patients resistant to fluorourea drugs have been studied in a phase 3 clinical trial. Also, there are two phase 4 clinical studies focusing on improving the prognosis of patients with COVID-19 infection by CsA combined with standard of care treatment, and on the efficacy of CsA to control HIV virus replication (Table 2). Another promising inhibitor is Alisporivir (Debio-025, C15), which has led to testing in phase 2/3 for use in combination with Peg-IFN and Ribavirin to treat chronic hepatitis C and inflammatory diseases. A recent clinical trial showed that in addition to Alisporivir‘s antiviral properties, it may also be effective in preventing lung tissue damage for the patients with infections due to SARS-CoV-2 (COVID-19) (Table 2). These studies suggest that cyclophilin A inhibitors are convincing immunosuppressant drugs for graft-versus-host diseases and inflammatory disorders.

Intriguingly, although CsA, the potent inhibitor of cyclophilin A, may induce skin cancer when used in transplantation [89,90,91,92,167,168,169], cyclophilin A has been observed to be upregulated in many solid cancers such as breast cancer, small-cell lung cancer, pancreatic cancer, colorectal cancer, squamous cell carcinoma, and melanoma [53]. Recent studies show elevated cyclophilin A expression in various cancers, promoting cell proliferation, migration/invasion, and apoptosis inhibition, with overexpression correlating with poorer patient outcomes [55]. Cyclophilin A upregulation has also been shown to confer resistance to cisplatin-induced apoptosis in several human cancer cells [170]. Similarly, an oligo-microarray analysis by Chen et al. [171] revealed that cyclophilin A can increase the expression of many cytokine-related, drug-transport-related, and drug-metabolism-related genes, which may lead to increased resistance of cancer cells to anticancer drugs. Although the underlying mechanisms of cyclophilin A on cancer development remain elusive, cyclophilin A inhibitors (especially CsA) have emerged for possibilities to treat human malignancies, including hematological and solid cancers, in clinical trials [78,79,172,173]. For example, despite the fact that CsA alone was ineffective for treating refractory colorectal cancer and produced significant toxicity [174], CsA in oral administration can modulate pharmacokinetics of irinotecan, the topoisomerase inhibitor. This insight is being used to alleviate toxicity in patients with fluorouracil refractory metastatic colon cancer [79]. A recent clinical trial revealed that in dose escalation cohort with advanced solid malignancies, CsA in combination with selumetinib, which involves the use of an MEK (mitogen-activated protein kinase/extracellular signal-regulated kinase) inhibitor, was well tolerated and showed evidence of antitumor activity in metastatic colorectal cancer [87]. Clinical studies support the hypothesis that cyclophilin A inhibitors could be promising in combination therapy for several human malignancies. 

Taken together, more and more promising evidence suggests that cyclophilin A inhibitors have been used in solid tumors in combination with established chemotherapeutic drugs, not just used as a potent immunosuppressants after transplantation in the patients with end-stage solid tumor or hematological diseases, but also as a direct therapeutic method for several solid tumors. The recent clinical trials not only affirmed the therapeutic potential of cyclophilin A inhibitors, but also highlighted their promising clinical application. These studies have enhanced researchers’ confidence in the development and approval of new drugs targeting cyclophilin A.

## 6. Conclusions and Perspectives

Cyclophilin A is recognized for its significant role in various biological processes and its association with numerous human disorders, such as inflammatory diseases and cancers, through its chaperone and peptidyl-prolyl isomerase (PPIase) activities. Its inhibitors have been discovered and characterized, and include the cyclic peptides (e.g., the first identified inhibitor CsA, SCY-635, and Alisporivir), the small molecule inhibitors (e.g., DC838), and the peptide analogues (e.g., WGP). In addition to CsA, several other inhibitors have entered clinical trials to assess their pharmacokinetics, efficacy, and safety. In addition to the classical roles of cyclophilin A inhibitors, plenty of clinical trials are focusing on the efficacy of the inhibitors in human hematological and even solid cancers. 

Since cyclophilin A has multifaceted roles in addition to immune response, there may be four future aspects and directions for the development of cyclophilin A inhibitors: (i) Identifying more potent inhibitors to target the PPIase activity of cyclophilin A effectively. It is a challenging and prospective direction to design novel inhibitors with anti-PPIase activity of cyclophilin A in the aspect of new technologies in drug design and discovery, such as PROTAC strategy [175], machine learning, artificial intelligence, quantum computing, and combined with existing computational drug design platform. (ii) Employing a diverse set of cyclophilin A conformations to identify and design the potential novel inhibitors. Accelerated molecules dynamics (aMD) has been applied to investigate the complex biomolecules. Considering the diverse functions of cyclophilin A in organisms, aMD is shown to be able to generate multiple of structures of a drug target, cyclophilin A [176]. These structures can be further used for structure-based computer-aided drug discovery and docking, and, thus, in the identification and design of potential novel inhibitors. (iii) Discovering nonimmunosuppressive inhibitors to advance the development of therapeutics for cyclophilin A-related cancers without compromising immune function. Research has shown that the expression of cyclophilin A is enhanced in HCC cells, and overexpressed cyclophilin A promotes HCC metastasis by upregulating matrix metalloproteinases MMP3 and MMP9 [57,177]. Therefore, one promising direction is to discover the inhibitors that can suppress the overexpression of cyclophilin A or the expression of MMP3 and MMP9, which, thus, will exclusively inhibit tumor growth but have no immunosuppressive effect. Recently, cyclophilin A short hairpin RNA, which has been identified as a nonimmunosuppressive PPIase inhibitor, can inhibit prolactin-stimulated signaling and regulate prolactin/Jak2-mediated tumor cell growth and migration [178]. This result may help us develop drugs for treating cancer based on cyclophilin A without interrupting immunity. (iv) Developing inhibitors that specifically target extracellular cyclophilin A through the cyclophilin A–CD147 complex. Previous studies have found that extracellular cyclophilin A stimulates cell proliferation by activating the ERK1/2 signaling pathway and CD147. Importantly, knocking down CD147 on hepatoma cells leads to a significant increase in T-cell chemotaxis by cyclophilin A induction both in vivo and in vitro [37]. These findings may provide a potential approach to discover novel cyclophilin A inhibitors to control cyclophilin A–CD147-related cancers.

However, it is important to note that many mechanistic details of cyclophilin A are still unknown and warrant further investigation., e.g., the fundamental roles of cyclophilin A in cancer development and progression, and the alternative receptors of extracellular cyclophilin A in addition to CD147. Moreover, many of the identified inhibitors are still under development. It is also urgent to discover novel and efficient candidate inhibitors of cyclophilin A to improve therapy regimen to reduce the toxicity and the off-target effects of the inhibitors themselves or the therapeutic drugs when used in combination. On the other hand, as CsA is also used in inflammatory diseases, finding nonimmunosuppressive CsA derivatives or new drugs that inhibit the role of cyclophilin A in inflammation and do not affect host immunity during viral infection control or cancer treatment still have a long way to go.

In summary, although the immunosuppressive agent CsA is well characterized, a wide range of cyclophilin inhibitors have emerged. These compounds have been proven to be effective against inflammation and cancer both in vivo and in vitro, and some are currently undergoing clinical trial evaluations. These advances have promoted the development of new drugs and encouraged further development of approved drugs, providing a promising strategy for treating inflammatory diseases and cancers. 

## Figures and Tables

**Table 1:** Structure of cyclosporin A and its analogues.

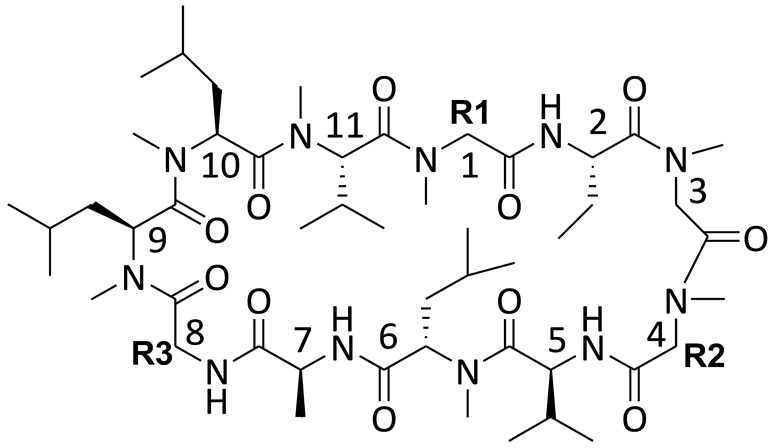 Cyclosporin A (CsA, C1)			
	**R1**	**R2**	**R3**
CsA	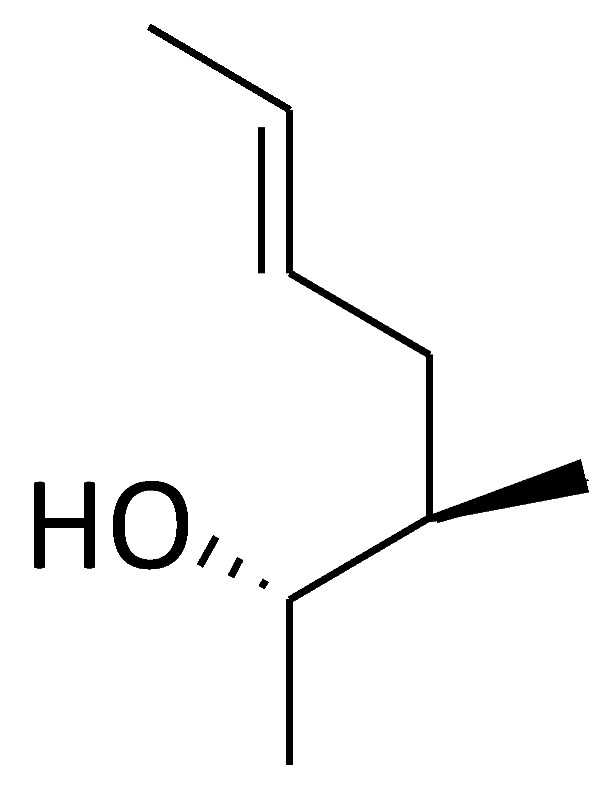	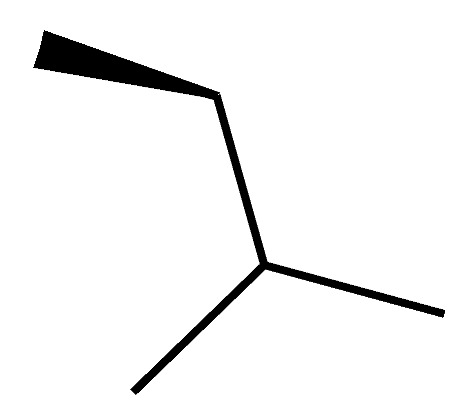	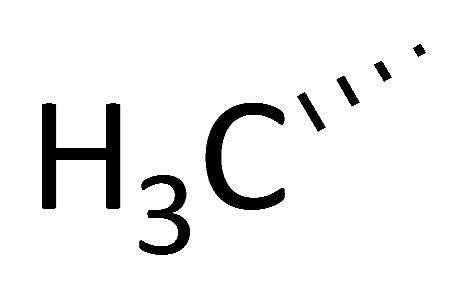
C8 (Dihydro-CsA)	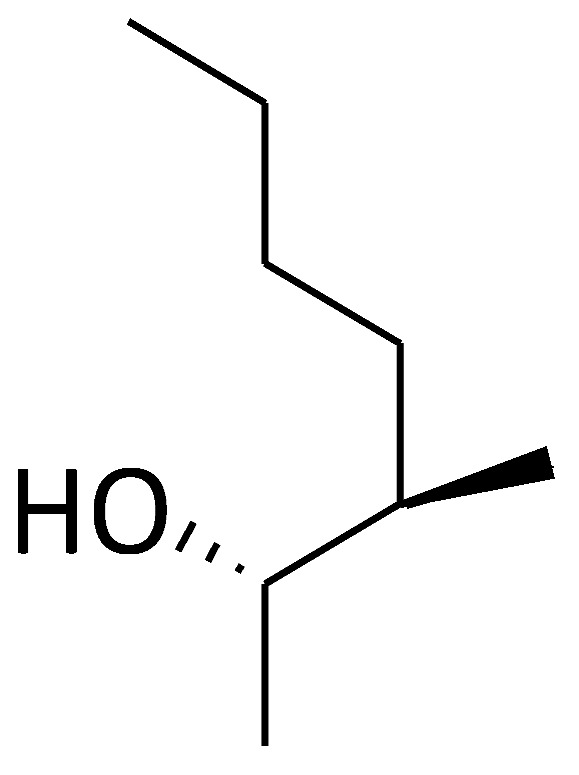	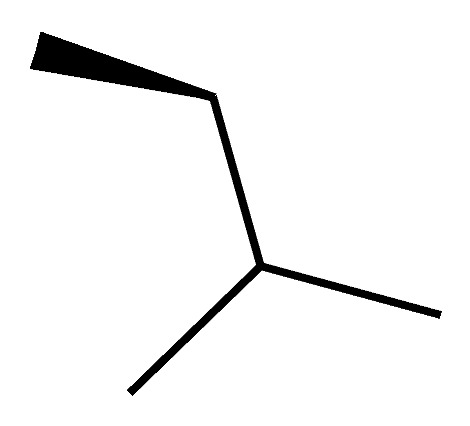	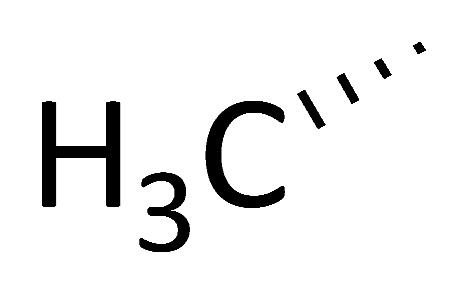
C9 ([DehydroAla]8-CsA)	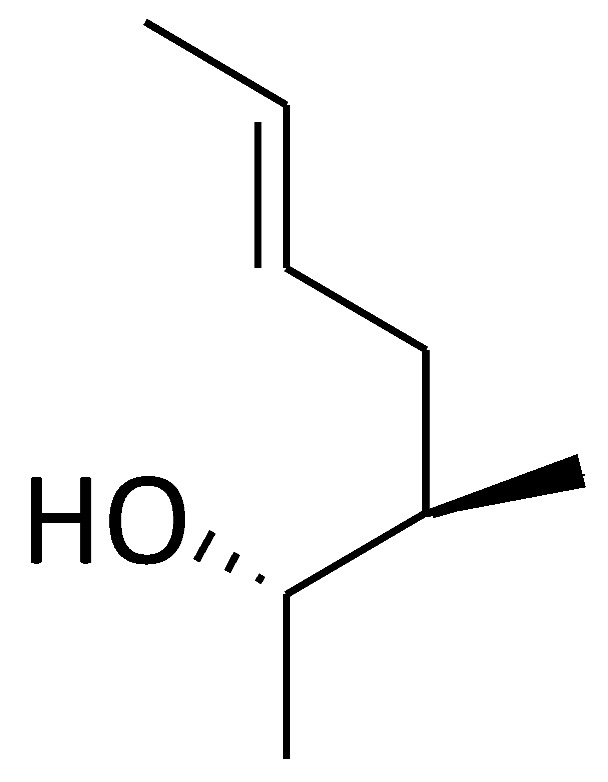	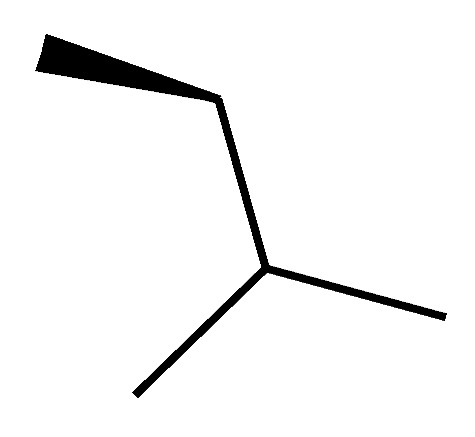	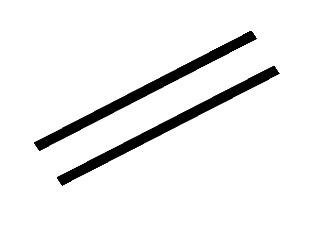
C10 ([MeVal]4-CsA)	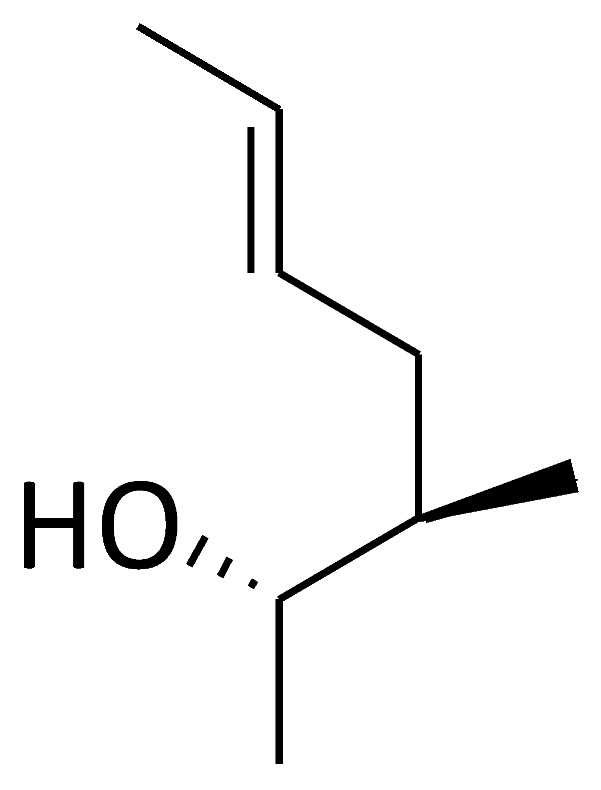	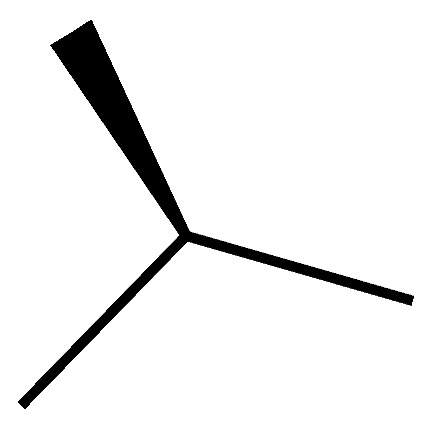	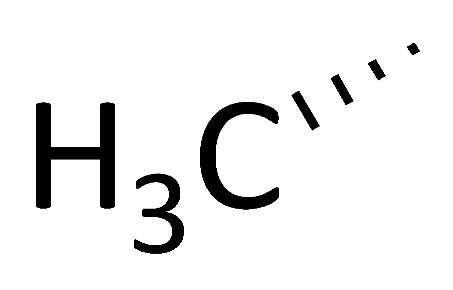
C11 ([MeAbu]4-CsA)	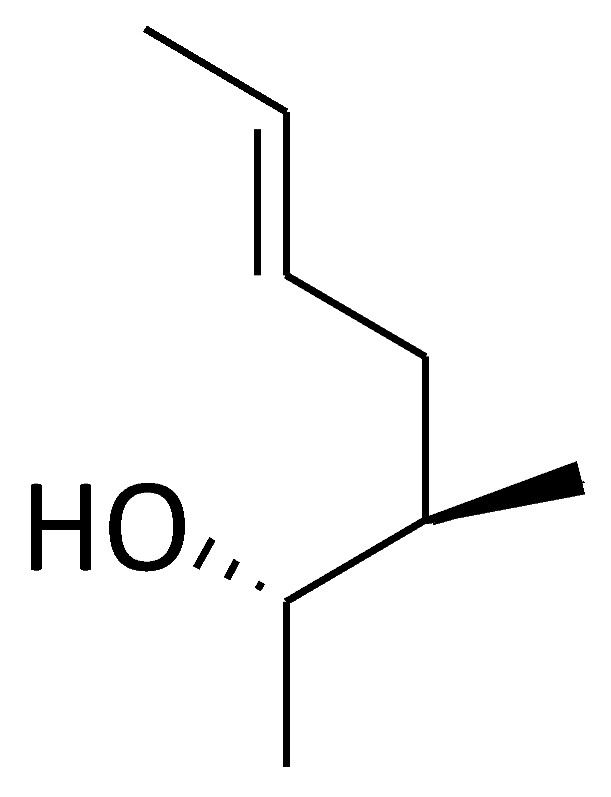	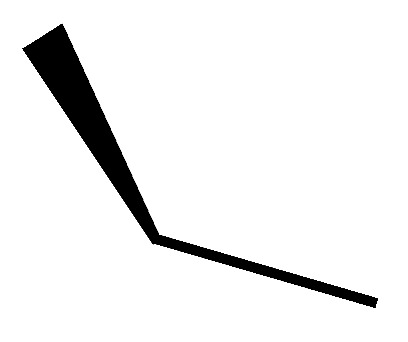	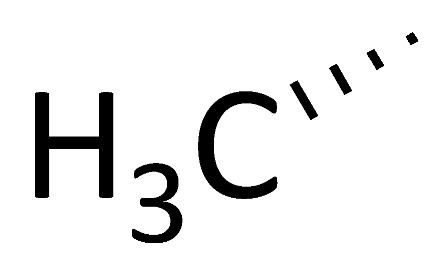
C12 ([Me(α-methyl)Thr]4-CsA)	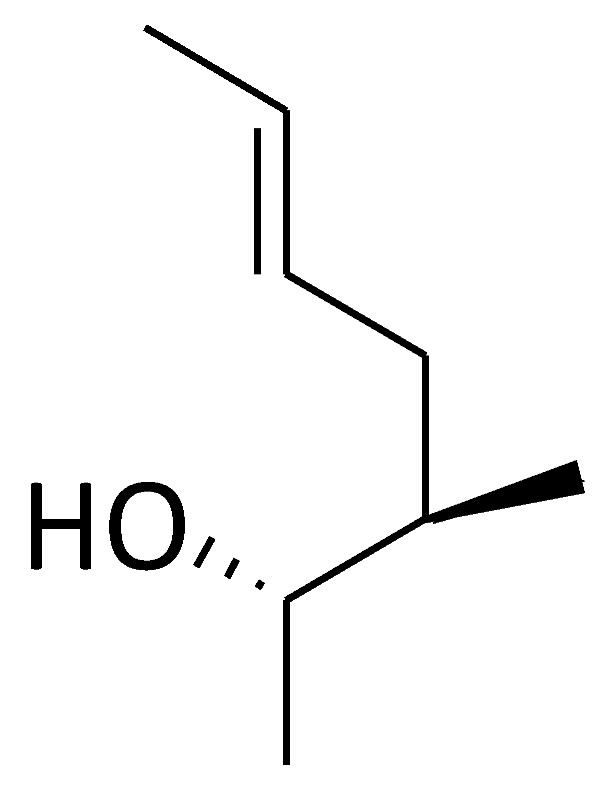	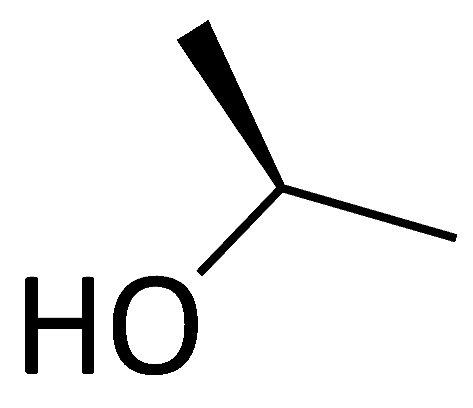	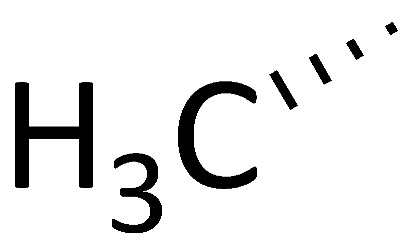

Note: R1, R2, and R3 are the positions for substituent groups. Numbers also show some key positions of carbon elements in cyclosporin A.

**Table 2:** Clinical trials of inhibitors of cyclophilin A ^a^.

Inhibitor Number	Inhibitor Name	NCT Number	Alone or in Combination	Sponsors	Diseases	Status
C1	Cyclosporin A (CsA)	~381 cancer-related clinical trials in early phase 1 and phases 1, 2, 3 or 4 ^b^	Alone or in combination	Virginia G. Kaklamani, Novartis, Allergan, NCI, MD Anderson Cancer Center, and others	Breast cancer, colon cancer, melanoma, nonmelanoma skin cancer, hematologic cancer, colorectal cancer, and others	Active, recruiting, completed, or terminated
		NCT00983424 (phase 1)	CsA Nab-paclitaxel	Northwestern University, Avon Foundation	Metastatic breast cancer	Completed
		NCT00003950 (phase 2)	CsA CPT-11	NCI, University of Chicago	Metastatic, advanced, or locally recurrent colorectal cancer	Completed
		NCT00389870 (phase 3)	CsA plus Irinotecan	University of Leeds	Colorectal cancer	Completed
		NCT04979884 (phase 3)	Alone	Alexandria University	COVID-19	Completed
		NCT04392531 (phase 4)	CsA plus SOC ^c^	Instituto de Investigación Sanitaria de la Fundación Jiménez Díaz	COVID-19	Completed
		NCT00979706 (phase 4)	CsA plus HAART ^c^	Hospital Clinic of Barcelona	HIV	Completed
		NCT00866684 (phase 4)	CsA as Comparator	Charite University, Berlin, Germany	Skin cancer	Completed
C13	SCY-635	NCT01290965 (phase 1)	Alone	Scynexis	Hepatitis C infection	Completed
		NCT01265511 (phase 2)	Alone	Scynexis	Hepatitis C infection	Completed
C14	NIM811	NCT00983060 (phase 2)	Alone	Novartis	Chronic hepatitis C Genotype-1 relapse	Completed
C15	Alisporivir (Deb 025)	NCT01975337 (phase 1)	Alone	Debiopharm International SA	Kidney failure	Completed
		NCT02173574 (phase 1)	Deb 025, EDP239	Enanta Pharmaceuticals	Hepatitis C infection	Completed
		NCT01860326 (phase 1)	Alone	Debiopharm International SA	Hepatitis C	Completed
		NCT01183169 (phase 2)	Deb 025, Peginterferon alfa-2a, Ribavirin	Debiopharm International SA	Hepatitis C infection	Completed
		NCT00537407 (phase 2)	Deb 025, Peginterferon alfa-2a, Ribavirin	Debiopharm International SA	Chronic hepatitis C	Completed
		NCT02094443 (phase 2)	Deb 025, Ribavirin	Debiopharm International SA	Hepatitis C infection	Completed
		NCT01215643 (phase 2)	Deb 025, Peginterferon alfa-2a, Ribavirin	Debiopharm International SA	Hepatitis C infection	Completed
		NCT04608214 (phase 2)	Alone	Assistance Publique—Hôpitaux de Paris	SARS-CoV-2	Completed
		NCT02753699 (phase 3)	Alone	DebiopharmInternational SA	Hepatitis C infection	Completed
		NCT01318694 (phase 3)	Deb 025, Peginterferon alfa-2a, Ribavirin	Enanta Pharmaceuticals	Hepatitis C infection	Completed

Note: ^a^, These data were retrieved from the ClinicalTrials.gov as of 28 February 2024. ^b^, There are >1000 clinical trials focusing on viral infection, transplantation, Sjögren’s syndrome, bone marrow failure, psoriasis, cancer, etc., with the status of active, recruiting, or completed. ^c^, SOC, standard of Care; HAART, highly active antiretroviral therapy.

## Data Availability

Not applicable.
